# Efficiency and Potential Evaluation to Promote Differentiated Low-Carbon Management in Chinese Counties

**DOI:** 10.3390/ijerph20043715

**Published:** 2023-02-20

**Authors:** He Zhang, Jingyi Peng, Rui Wang, Yuanyuan Guo, Jing He, Dahlia Yu, Jianxun Zhang

**Affiliations:** Department of Urban Planning, School of Architecture, Tianjin University, Tianjin 300072, China

**Keywords:** China, county, low-carbon management, efficiency, potential, zoning

## Abstract

Low-carbon management plays an important role in mitigating climate change and adapting to it. Localities should adopt differentiated low-carbon management policies according to the state of their environment. To help formulate specific and realistic low-carbon management policies, this paper took into account specific low-carbon management sectors. Likewise, it carefully considered the differences in various resource endowments and proposed a method for evaluating low-carbon management efficiency and potential. The method was applied to an empirical study from 2015 conducted on 1771 Chinese counties. Significant spatial heterogeneity was found during the research. The counties bordering central and Western China and the ones in the southeast coastal areas showed higher efficiency in the industrial sector. Southern and Northern China had higher efficiency in the housing and transportation sector, respectively. Moreover, counties in remote areas showed more potential in the industrial sector. Central China had higher potential in the housing sector, while counties bordering provinces had more potential in the transportation sector. Therefore, Chinese counties were divided into eight management zones where differentiated management strategies were identified to shape low-carbon management policies.

## 1. Introduction

With the rapid melting of polar glaciers and rising sea levels, global warming has become a major concern for the international community. Reducing greenhouse gas emissions and curbing global warming has become a common goal for society in this century [1]. Among the greenhouse gases, the carbon emitted through human activities is the most common cause of global warming [2]. Therefore, the Paris Agreement was adopted with the growing awareness of this threat. In particular, the parties set binding national targets for reducing carbon emissions. Notably, China, as the largest carbon emitter in the world [3], had pledged that its carbon emissions will have peaked and balanced out by 2030 and 2060, respectively [4].

In order to achieve the national reduction target, China needs joint efforts at a local level. Apart from technology upgrading, low-carbon management is also significant [5,6]. Low-carbon management is an organized approach to gain the strategic advantages of carbon emission reduction. It is believed to play an important role in mitigation and adaptation co-benefits [7,8]. Scientific low-carbon management can reduce industrial and household energy waste, improve energy use efficiency, and actively guide low-carbon behavior and habits in industrial and residential areas [9,10]. When it comes to specific management, it is worth noting that different regions have different resource endowments and development conditions [11]. As a result, it is impossible to require all localities to adopt the same low-carbon management objectives and strategies, so each region needs a differentiated management method. Efficiency evaluation can be used to assess the actual environmental situation, while potential evaluation can show the gap between the target and the actual situation. Efficiency and potential evaluation take into consideration the realistic regional conditions and the difference in potential and help in judging the difficulty and possibility of carbon control adjustment and putting forward more reasonable low-carbon management policies. Therefore, it is necessary to establish a detailed and systematic method to evaluate the efficiency and potential of low-carbon management and then formulate low-carbon management goals and paths for different regions in China.

In the study of differentiated low-carbon management, Chinese counties are particularly worth attention. The county is an important and basic governance level. In contrast to the city district, the county is located in a more marginal area and governs towns and villages. Counties account for 88% of the land area, 74% of the population, and 60% of total CO_2_ emissions in China, and they are of great significance for China to achieve carbon neutrality [12]. In addition, low-carbon management research is urgent at the county level. As the key area connecting urban and rural areas, county management is complex and important [13]. However, due to the inadequate development conditions and management ability of the counties, the improvement of the management level is still inadequate. Moreover, compared with cities and regions, counties are smaller in size and face a more specific management scenario. Most of the experiences of carbon management in typical large cities are not suitable for directly applying to the counties, and special research is needed. Furthermore, Chinese counties have the necessity and representativeness of differentiated management research. They exist obvious differences in resource endowments and development conditions. For example, county units are widely distributed in mountainous, plateau, plain and other terrain areas in China, and the climate distribution also covers warm and cold regions. In terms of the level of economic development, the development level of the top 100 counties, such as Kunshan County, has reached the level of developed countries, while the problem of poverty in some counties still exists. Therefore, it is necessary to assess the efficiency and potential of low-carbon management in Chinese counties so as to help conduct differentiated low-carbon management in China.

Numerous studies researched low-carbon cities, counties and low-carbon management and put forward a series of low-carbon management indicators for specific energy consumption sectors [14,15,16]. These studies have laid the foundation for formulating low-carbon management paths in Chinese counties. Likewise, some studies further identified the main drivers of carbon emissions in different regions [17,18,19], as well as carrying out the low-carbon development assessment in various regions [2,20], which can clarify the possible and important policies of low-carbon management and help differentiate them. Efficiency and potential evaluation can further clarify the current state of the management and also reveal its possible promotion, which is of great significance for further research on differentiated low-carbon management. However, relevant research does not involve the county level, and the studies in provinces and cities just calculated the overall managerial inefficiency based on the potential technological heterogeneity [21,22,23]. Such studies can help set an overall management goal but cannot help formulate a specific implementation path since low-carbon management involves different energy consumption sectors and management elements. There are a few pieces of research that consider them and investigate their efficiency and potential to clarify which key sectors need improvement. Furthermore, the existing research considered local differences in technological heterogeneity and disregarded numerous differences in resource endowments between regions. In fact, resource endowments vary greatly among regions, especially at the county level. If these barriers in management improvement are not set, the calculation results and management objectives may be unrealistic.

This paper aims to establish a management efficiency and potential evaluation method to support the differentiated management of Chinese counties from the perspective of specific management promotion. The method intends to involve management sectors and indicators to determine specific management, as well as consider the gap in resource endowment to guide feasible promotion. To achieve this, the paper aimed to answer three main research questions: (1) How to introduce low-carbon management sectors and indicators to an efficiency evaluation system so as to evaluate the management efficiency of each low-carbon management sector; (2) Considering the various resource endowments, what is the most feasible efficiency target and the corresponding potential of key management sectors for each county; and (3) Based on the efficiency and potential evaluation results, what differentiated low-carbon management path should Chinese counties take?

This study contributes to its respective field in the following ways: (1) In different counties, specific low-carbon management sectors were evaluated to clarify key elements that need improvement. The sectors then helped formulate more specific policies; (2) Differences in resource endowments were considered to clarify the barriers to management improvement and determine the efficiency target and management potential of each county, which can help formulate reasonable low-carbon management goals and reference examples; and (3) This paper proposed differentiated low-carbon governance zones for Chinese counties, which help the country implement macro policy control and persuade local governments to formulate specific and feasible low-carbon management measures according to local conditions.

The rest of this paper is organized in the following way. Section 2 reviews the related literature. Then, Section 3 describes the methodology in detail. Section 4 presents the results and discusses the empirical study. Lastly, Section 5 provides key findings and discusses the limitations of this paper.

## 2. Literature Review

### 2.1. Research on Low-Carbon Management Elements

In the past few decades, scholars have conducted research on low-carbon cities, counties and influencing factors that affect carbon dioxide emissions. Elements of low-carbon management support and specific policy management indicators have been discussed in the context of major energy consumption sectors such as the industry, housing, and transportation [24,25,26].

In the industrial sector, management indicators reflecting industrial structure, such as the proportion of the tertiary industry to gross domestic product (GDP) and the structure of the industry in economic development, proved to have a significant impact on carbon emissions and efficiency [27,28,29,30]. Similarly, management indicators reflecting asset investment, such as gross fixed capital formation (GFCF), are considered to be closely related to carbon emissions [31,32]. In the housing sector, management indicators expressing development intensity, such as population density, were found to correlate with carbon emissions in residential areas [33,34,35]. The construction of infrastructure services relates to heat, gas, electricity, and other supply for heating, daily cooking, lighting, and household appliances that impact energy consumption in residential buildings [36,37]. In the transportation sector, studies found that road planning and construction and pedestrians are considered to cause carbon emissions [38,39,40]. In addition, the organizational and spatial layout, such as the construction of public service facilities, can affect transportation modes and travel distances, thereby producing more carbon emissions [41,42,43,44].

As mentioned above, existing research provided numerous elements and indicators of low-carbon management, which can help carry out specific low-carbon management measures.

### 2.2. Research on Differentiated Low-Carbon Management

It is worth noting that methods of low-carbon management should be different between regions due to their heterogeneity. In order to achieve the national emission reduction target, differentiated management policies must be adopted for each region. The existing regional differentiation studies related to carbon emissions mainly described the spatial distribution pattern of carbon emissions and their efficiency [45,46,47,48]. However, these descriptions cannot reflect the internal mechanisms and problems that lead to carbon emissions and, consequently hard to create differentiated low-carbon management.

To differentiate low-carbon management, some research further studied the regional differences in the driving factors of the carbon emissions [18,49,50,51,52,53]. For example, Li et al. (2017) analyzed the provincial differences in the driving factors of carbon emission [54]. Li et al. (2022) explored the different effects of socioeconomic and urban form factors on carbon emissions in Chinese cities [19]. Based on differentiated key driving factors, Zhang et al. (2021) studied the carbon emission governance zones in Chinese counties [13]. In essence, these studies can reflect the influence of factors on carbon dioxide emissions and identify key driving factors in different regions. However, in view of the differences in the development needs, as well as the wide differences between regions. The regions with strong carbon dioxide emission drivers are not necessarily the regions that need the most management improvement, and the main driving factors of each region are may not the factors that need the most attention for management improvement.

At the same time, other studies have conducted differentiated low-carbon management research in the form of low-carbon development evaluation. For instance, Du et al. (2018) evaluated the level of low carbon development in 30 provinces in China from 2003 to 2013 based on the maximum flux principle [20]. Wang et al. (2021) evaluated the quality of low-carbon development of 259 cities in China according to the score of low-carbon development quality calculated by the TOPSIS method [2]. However, fruitful insights have been made into the evaluation of low-carbon development, which has provided important references for low-carbon management. However, relevant research at the county level has not been carried out. Furthermore, previous studies mostly evaluate and analyze low-carbon development status without further exploring the management improvement space affecting the development status. Therefore, it is hard to propose targeted low-carbon optimization policies.

### 2.3. Research on Efficiency and Potential Analysis

Efficiency and potential analysis are often used to measure carbon emission efficiency and carbon reduction potential. There has been a large number of research focused on the nation, province and city scale. For example, Li et al. (2021) used a non-radial directional distance function to determine the efficiency of carbon dioxide emissions and potential emission reduction of States parties to the Paris Agreement [22]. Zhang et al. (2016) evaluated the industrial carbon dioxide emission efficiency and emission reduction potential of 30 provinces in China from 2005 to 2012 [55]. Liang et al. (2020) estimated the CO_2_ emission efficiency of 13 cities in Beijing, Tianjin and Hebei in 2005–2014 by using the unexpected output SBM model and further made quantitative analysis of carbon emission reduction potential based on the fairness and efficiency of the emission reduction potential index [56].

In recent years, with the emphasis on management, the analysis of low carbon management has also begun to include the evaluation of carbon emission efficiency and carbon emission reduction potential [57,58,59]. For example, Yao et al. (2015) and Sun et al. (2018) calculated the technical and managerial inefficiencies of energy use on the regional and city scale across China [23,60]. They based their research on the data envelopment analysis (DEA) model and the carbon emission reduction potential calculated using the meta-frontier method. These studies can clarify the importance of management improvement in carbon control and emission reduction in various regions and help each region propose differentiated low-carbon management goals. However, there is no relevant research on the county level. Moreover, low-carbon management involves energy consumption sectors such as the industry, housing, and transportation, so a variety of specific management improvement methods is needed. Existing research can help formulate the goals but cannot clarify the key points of low-carbon control and offer each region opportunities to improve.

Some individual studies involved specific management ideas and calculated the impact of specific management factors on the carbon emission reduction potential in different regions [61,62]. For example, Yu et al. (2015) used two independent variables, i.e., the GDP and the proportion of the tertiary industry to GDP, and estimated the effect of the variables on carbon abatement potentials [62]. However, most studies focused on the management elements of the industrial sector, ignoring the potential roles of other sectors and other low-carbon management elements. Thus, the studies could not form low-carbon management goals.

In addition, the formulation of management strategies should be not only specific but also realistic. Heterogeneity among regions can have an impact on carbon control and emission reduction. For example, Wang et al. (2015) analyzed Chinese cities and concluded that high- and low-earning cities had higher energy conservation efficiency, while middle-earning cities had the lowest [63]. Zhang et al. (2021) found that the carbon emission characteristics of Chinese counties were closely related to heating, economic and population density, and topographical zoning [13]. In fact, differences in regional economic development and topographic and climate conditions have limited the implementation and improvement of low-carbon management policies. Low-carbon management must consider the differences in resource endowments in order to formulate realistic control strategies. However, existing studies only considered technological heterogeneity [21,55,60,64]. There are still other resource endowments that affect carbon emissions and limit the improvement of low-carbon management.

To fill this research gap, this paper intends to take counties in China as the research object and propose an efficient and potential evaluation method for low-carbon management so as to promote differentiated carbon management. This goal can be divided into two aspects: (1) Switch the research perspective of differentiated low-carbon management from status evaluation to optimization policy. Specifically, from the analysis of driving factors impact and evaluation of low-carbon development status to the management improvement based on management efficiency and potential evaluation. (2) Deepen the relevant efficiency and potential research and focus on low-carbon management. On the basis of overall managerial inefficiency analysis in existing research, further, introduce the specific low-carbon management sectors and elements, as well as comprehensively consider the difference in resource endowment between counties so as to meet the needs of differentiated low-carbon management. The goal is based on the following hypotheses: Considering the differences in local realities of Chinese counties, the further analysis of management efficiency and potential will show obvious regional heterogeneity to guide differentiated low-carbon management, and the analysis result can be different from that of driving impact and status evaluation.

## 3. Methodology

### 3.1. Data Sources

Based on available data, the cross-sectional data from 2015 was selected to research 1771 out of 1991 Chinese counties. The Tibet area and a few others were not included in the study due to a lack of data. The carbon emission data were derived from the high spatial resolution grid data of carbon emissions in China. The grid data was standardized by GIS spatial analysis to obtain the carbon emission data of Chinese counties in 2015. The management variables data were taken from the *2015 Chinese counties Construction Statistical Yearbook* and *the 2016 Chinese counties Statistical Yearbook*. Although the research data is relatively old, Chinse counties are different from city districts, and their low-carbon management process is lagging behind. The results of the research still have important reference significance.

### 3.2. Efficiency Evaluation through the DEA Method

This research established a sub-sectoral low-carbon management efficiency evaluation system and evaluated the low-carbon management efficiency of the industrial, housing, and transportation sectors. In turn, it helped in judging the efficiency of regional low-carbon management, evaluating the effectiveness of key development areas, and analyzing where deficiencies exist. The DEA method is good at dealing with the efficiency problem of multiple input and output factors. It utilizes the relative efficiency evaluation method and is often used for comparative research in multiple regions, which is highly applicable to this study.

The DEA evaluation system takes the low-carbon management variables and effects as the input and output elements, respectively. In order to determine the county-level low-carbon management variables as input variables, this study refers to the typical variables of low-carbon cities, counties and influencing factors that affect carbon dioxide emissions published in international and Chinese journals [2,12,13,65,66,67,68,69]. On the basis of available data, we select the variables with significant correlation with carbon emission efficiency and remove the repetitive variables. Finally, 11 variables shown in Table 1 were selected. These variables include various aspects of low-carbon management, such as industrial structure, asset investment, development intensity, municipal utility facilities, road traffic facilities, and functional space layout. It is worth noting that the external traffic of counties is mainly dominated by roads, and the external transportation of railway, ships and aircraft is very few, which is not considered in the study. In addition, the traffic indicators related to residents’ rigid travel needs, including the allocation of schools and medical facilities, deserve attention in the low-carbon management of the county. For output elements, the GDP output per unit of industrial carbon emission, the residential population per unit of housing carbon emission, and the coverage area per unit of carbon emission from transportation was selected. The final input-output elements of the DEA model are shown in Table 1.

The basic principle of DEA is to use linear programming to find the production frontier of the decision-making unit. Only the decision-making unit that falls on the boundary has an efficiency value of 1, called the DEA effective, while the DMU that does not fall on the boundary has an efficiency value between 0 and 1, called the DEA invalid. The CCR model proposed by Charnes et al. (1979) was used for calculation [70]. The model assumes that n decision-making units exit (DMU_i_, i = 1,2, …, n). Each unit has m inputs and s outputs. X_i_ = (x_1i_, x_2i_, …, x_mi_)^T^, Y_j_ = (y_1j_, y_2j_, …, y_sj_)^T^, where x_ij_ is input i of unit j, while y_rj_ is output r of unit j. v = [v_1_, v_2_, …, v_n_]^T^, (v ≥ 0) is the weight vector of the inputs, and u = [u_1_, u2, …, u_n_]^T^, (u ≥ 0) is the weight vector of the outputs.

The efficiency index of DMU_j_ is

(1) $$h_{j}=\frac{\sum_{r=1}^{s}{u_{r}y}_{rj}}{\sum_{i=1}^{m}{v_{i}x}_{ij}}.$$

The CCR model for evaluating the efficiency of DMUj0 is:

(2) $$\{\begin{array}{c} \min[\theta -\varepsilon (e_{1}^{T}s^{-}+e_{2}^{T}s^{+})]=V_{D}\left(\varepsilon \right)\\ s.t.\sum_{j=1}^{n}x_{j}\lambda_{j}+s^{-}=\theta x_{0}\\ \sum_{j=1}^{n}y_{j}\lambda_{j}-s^{+}=y_{0}\\ \lambda_{j}\geq 0,j=1,2,...,n\\ s^{-}\geq 0,s^{+}\geq 0 \end{array}$$

In Equation (2), θ refers to the input ratio variable; ε refers to the non-Archimedean infinitesimal variable, usually valued 10^−6^; e_1_^T^ = (1, 1, …, 1) ∈ E_m_, e_2_^T^ = (1, 1, …, 1) ∈ E_s_^−^, s^−^ = (s_1_^−^, s_2_^−^, …, s_m_^−^) is the slack variable vector corresponding with inputs; and s^+^ = (s_1_^+^, s_2_^+^, …, s_s_^+^) is the residual variable vector corresponding with outputs.

### 3.3. Potential Evaluation through the Efficiency Variance Method

In existing research, potential evaluation methods are divided into three categories: the efficiency variance estimation method, the scenario analysis estimation method, and the econometric energy model. The scenario simulation method and econometric energy model are used in researching a single region, while the efficiency variance estimation method is used in comparative studies of multiple regions. This study used the efficiency variance method to calculate the potential of low-carbon management in Chinese counties. The management efficiency of county i is set as β_i_. The calculation result with the highest efficiency value was taken as the reference value, namely β_max_. The low-carbon management potential Q_i_ of other counties is:

(3) $$Q_{i}=1-\frac{\beta_{i}}{\beta_{max}}$$

The potential of each county implies the absolute convergence of the maximum efficiency value of each county and the frontier object. In order to obtain precise potential calculation results for low-carbon management practices, the most important thing is to select appropriate low-carbon frontier reference objects for each county. Thus, this research considered a variety of resource endowments for grouping regions to ensure that the identified frontier objects have a realistic reference.

The level of economic and technological development, topography, climate conditions, and the residents’ income levels should be considered in the differential analysis of resource endowments. In the industrial sector, the level of economic and technological development determines the development stage and technological level of the industry. The level of economic and technological development is often insurmountable, so regions with different levels of development exhibit great differences in the low-carbon management of the industrial sector. In the housing sector, residents’ income levels and climate and topographic conditions impact carbon emissions from housing. These conditions should be considered as the grouping factors of the construction sector. Specifically, great differences are found in the consumption of domestic energy in different climate zones. Likewise, the corresponding configuration standards of municipal facilities are different. Housing energy consumption is closely related to the residents’ income level. In areas with different income levels, significant differences are found in the resident’s energy consumption habits. In addition, topography can affect the configuration of municipal utility facilities that also impact domestic energy consumption. In the transportation sector, residents’ income levels and topography can significantly affect transportation modes, the difficulty of road construction, and the functional space layout, and should be seen as grouping factors. The grouping factors for each management sector are shown in Table 2.

Based on the grouping factors, it is necessary to divide counties into groups to ensure that the ones in the same group share similar resource endowment conditions while the others set realistic barriers difficult to overcome. Based on theory, the groups were classified in the following way. Eight comprehensive economic zones proposed by the Development Research Center of the State Council were used to divide the economic and technological development level of the groups. Then, six geomorphological zones proposed by the Chinese Academy of Sciences were used to divide the topographic group. Seven climate zones in the Chinese architectural division were used to divide the climate zones. Lastly, four development stages proposed by the World Bank were used to divide the residents’ income levels.

## 4. Results and Discussions

### 4.1. Efficiency Evaluation Analysis

The low-carbon management efficiency of the industrial, housing and transportation sectors in Chinese counties was measured through the efficiency evaluation method. The low-carbon management efficiency of Chinese counties was low, while the average efficiency of each sector was lower than 0.1. In particular, the average management efficiency of the transportation sector was the highest, while the housing sector had the lowest average efficiency. Regarding the standard deviation of management efficiency, the standard deviation of the housing sector was the lowest, while that of the transportation sector was the highest. The low-carbon management efficiency of the transportation sector greatly deviated from the average across all counties, and the management level varied from place to place.

The evaluation results were displayed in GIS to clarify the distribution of low-carbon management efficiency in Chinese counties. The counties were divided into six sections: high efficiency, medium–high efficiency, medium efficiency, medium-low efficiency, and low-efficiency areas. To make the pattern clear, the quantile method was used for grading division, ensuring that the numerical interval changed uniformly between classes and that each class had a certain number of objects.

**Figure 1:**
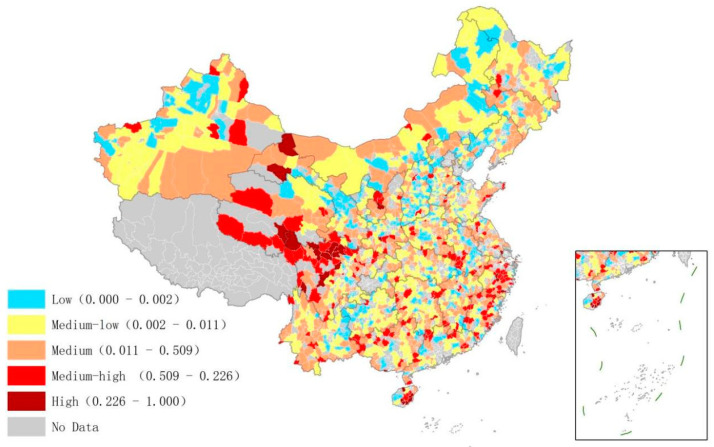
Low-carbon management efficiency of the industrial sector in Chinese counties.

The distribution of low-carbon management efficiency in the industrial sector is shown in Figure 1. The overall efficiency across all counties was low. Among the 1771 counties, 994 were classified as having low or medium-low efficiency. Areas with higher efficiency accounted for a small proportion. These areas were mainly located in the central and western provinces and the southeastern coastal provinces, such as Southern Qinghai Province, Northern Sichuan Province, Eastern Hainan Province, and southeastern coastal provinces Jiangsu and Zhejiang. The analysis found that the higher efficiency was either located in remote areas dominated by green industries such as tourism or in counties with high economic development and a mature management level. For example, Kangding County in Sichuan Province as well as Qionghai County in Hainan Province, which is dominated by the green industry, have high efficiency in the industrial sector. Kunshan County in Jiangsu Province and Yiwu County in Zhejiang Province, which with high economic development, also have high efficiency.

**Figure 2:**
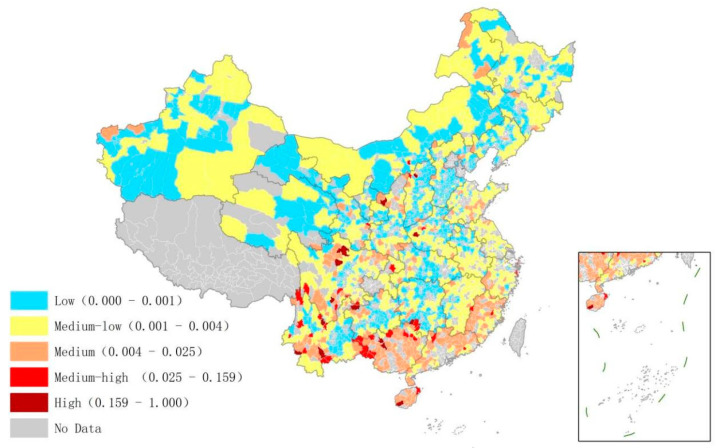
Low-carbon management efficiency of the housing sector in Chinese counties.

Figure 2 shows the distribution of low carbon management efficiency in the housing sector. The result shows that the management efficiency in the south was generally higher than that in the north of China. This may be related to the difference in heating policies between the north and the south. At the same time, the overall efficiency of the housing sector in Chinese counties was extremely low. A total of 1464 of 1774 counties were found in low or medium–low efficiency areas. Only 14 counties showed high efficiency, mainly in the Yunnan, Sichuan, and Guizhou provinces. These areas are relatively economically backward and have fewer demands for energy consumption. However, they show certain characteristics of population agglomeration and relatively good infrastructure.

**Figure 3:**
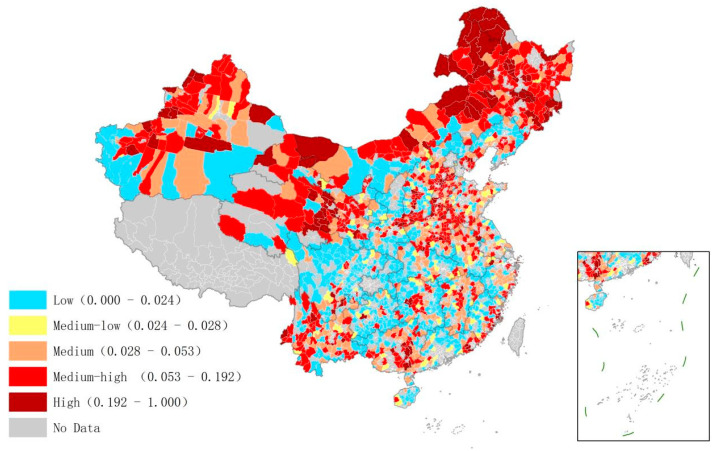
Low-carbon management efficiency of the transportation sector in Chinese counties.

The distribution of management efficiency in the transportation sector is shown in Figure 3. The overall management efficiency of the transportation sector in the north was higher than that in the south of China. This may be because China has established relatively unified national standards for road construction and public service facility development, so there was little difference in construction management between the north and the south. However, the northern region has vast plains and fewer mountains and hills. Thus, the transport efficiency was high, which improved the management efficiency.

### 4.2. Potential Evaluation Analysis

Based on the calculations of the low-carbon management efficiency of each sector, the Chinese counties were grouped in regard to various resource endowments. Likewise, the efficiency variance method was used to calculate the potential. Overall, the average potential of the industrial sector was the highest, slightly higher than that of the transportation and housing sectors. The average potential of the transportation sector was found to be very close to that of the housing sector. It is worth noting that the average management potential of the three sectors reached more than 0.85, indicating that each sector can improve its management. In addition, the standard deviations of potential in the sectors were not very different. The industrial sector had the smallest standard deviation with small differences in emission reduction potential across regions.

**Figure 4:**
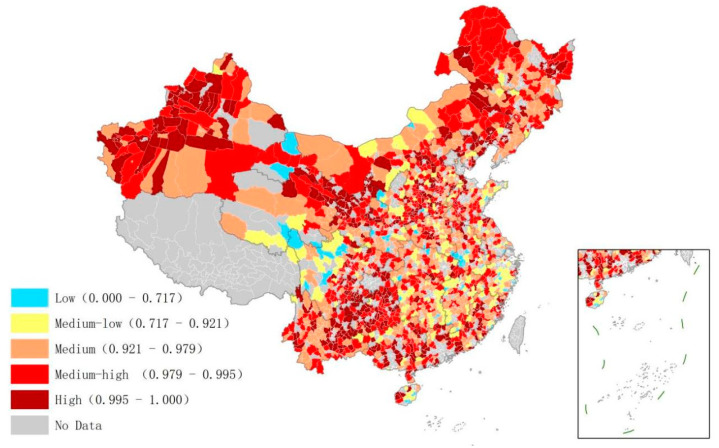
Low-carbon management potential of the industrial sector in Chinese counties.

In order to clarify the distribution of low-carbon management in Chinese counties, this paper adopted the quantile classification method to display the management potential in GIS. The distribution of low-carbon management potential in the industrial sector is shown in Figure 4. High management potential was mainly distributed in remote areas in central and Western China, such as Xinjiang, Yunnan, and Guizhou provinces. It also showed a spatial agglomeration state in areas such as western Xinjiang, eastern Yunnan, and central Shaanxi provinces. Areas with a high potential generally showed high carbon levels due to economic development. The economic development of the mentioned areas relies heavily on the secondary industry. This industry is still underdeveloped because it has low technological content, weak technological innovation capabilities, and heavy industries with high energy consumption that cause pollution. However, it shows signs of growth. Similarly, the tertiary industry in these areas is underdeveloped. In contrast to the secondary industry, it is developing slowly. The industrial and economic structure is still unstable in these areas, creating room for improvement in low-carbon management. On the contrary, the eastern coastal areas and Northwestern Sichuan and Southern Qinghai provinces showed less potential. This can be attributed to the fact that either the secondary industry in these areas has high technological content and development or that they mainly rely on the development of the tertiary industry. Therefore, there is relatively little room for improvement in low-carbon management in such areas.

**Figure 5:**
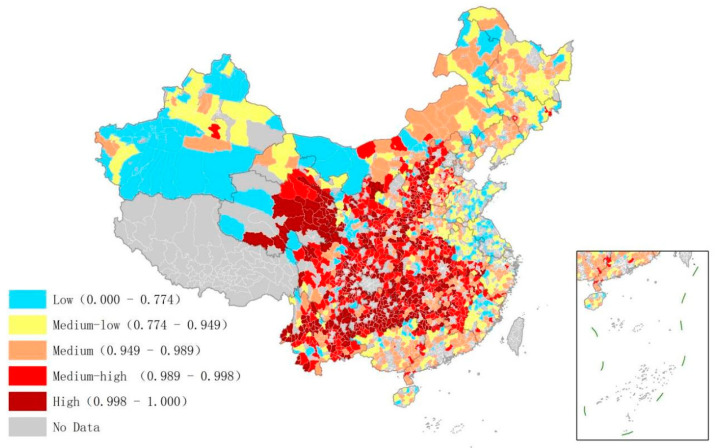
Low-carbon management potential of the housing sector in Chinese counties.

The spatial distribution of low-carbon management potential was clustered in the housing sector (Figure 5). High management potential was mainly distributed in central China, while the relatively low potential was found in the eastern coastal area and Northwestern China. High potential in central China can be attributed to the low level of municipal facility construction in comparison to more advanced areas. Likewise, the management of energy used for housing in these regions was relatively weak. Due to the improvement in living standards, residents corresponded a preference for large houses and habits of high energy consumption, which cause higher carbon emissions, proving that the low-carbon management efficiency in the housing sector is far from that in the advanced areas and that it needs to be urgently improved.

**Figure 6:**
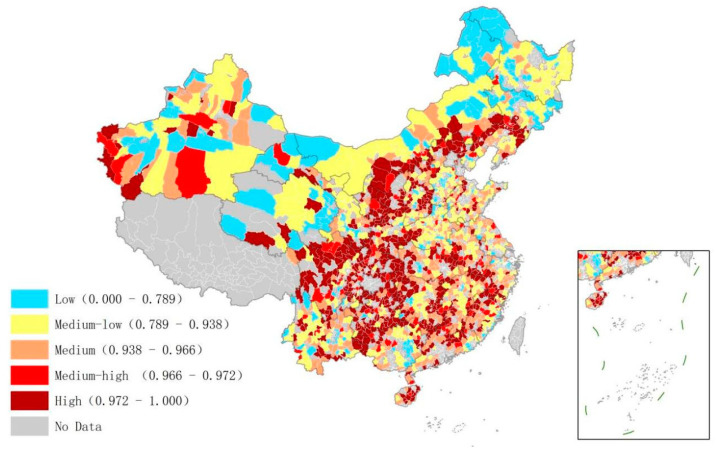
Low-carbon management potential of the transportation sector in Chinese counties.

Figure 6 shows the distribution of low-carbon management potential in the transportation sector. The high potential was mainly distributed in junctions between provinces, including the area between Hunan Province and Jiangxi, Guangdong and Guizhou Province, Guangxi and Guizhou Province, and Inner Mongolia and Liaoning Province. On one hand, the provincial bordering areas often pay less attention to planning, construction, and management, attributing to high potential. There are many reasons for this. Firstly, the bordering areas are often far from the central, provincial cities, and their economy is less developed, resulting in insufficient funds and capacity to support construction and management. Because most of the bordering areas are mountainous, construction is difficult and expensive, complicating the implementation of infrastructure. Furthermore, cross-border administrative management is poor due to the Chinese regional management system, causing incomplete and disconnected roads and public service facilities. All these factors lead to a significantly lower level of construction and management in border areas. On the other hand, bordering areas often have more developed cross-border transportation and travel due to logistics transportation. The backward supporting management policy and high frequency of travel inevitably lead to extremely low management efficiency, so urgent improvement is required in these areas.

### 4.3. Differentiated Governance Zones and Paths

**Figure 7:**
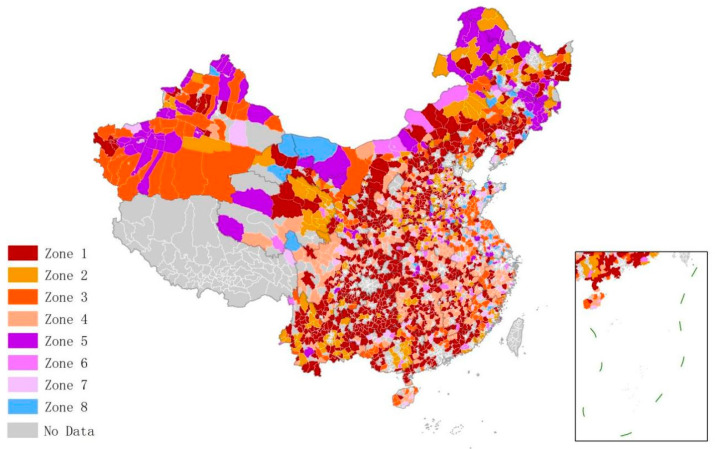
Differentiated governance zoning map of Chinese counties.

Counties in different regions should adopt differentiated policies according to their conditions of the environment. Chinese counties were divided into eight governance zones according to their main potential sources (Figure 7). Key control sectors in each zone were different. According to the number of key control sectors in each zone, this paper adopted four different policy orientations. The governance paths for each zone are shown in Table 3.

The management potential of the industrial, housing and transportation sector was higher than the average in Zone 1. Thus, it represents the key area that needs to improve its low-carbon management. A total of 760 counties were found in this zone, accounting for 42.7% of the 1771 counties. This shows that nearly half of the Chinese counties need to recognize the important role of low-carbon management in carbon control and emission reduction and further formulate active policies. Based on spatial distribution, counties in Zone 1 are mainly located in the central part of China. In order to improve low-carbon management efficiency, these counties should make breakthroughs and substantially improve their low-carbon management levels in each sector.

Zones 2, 3, and 4 had two sectors with above-average management potential. The three zones included 746 counties, accounting for 42.2% of the 1771 counties. These areas are important and need to improve low-carbon management. Coordination of low-carbon management efficiency should be improved in the two sectors with higher potential, while management of the sector with lower potential should be consolidated. Zone 2 consists of Yunnan, Qinghai Province, and other areas. Here, it is necessary to pay attention to the low-carbon management of the industrial and housing sectors. Zone 3 spreads across the Xinjiang region, where the key control sectors are the industrial and transportation ones. Zone 4 constitutes the central region of China. It should focus on strengthening the low-carbon management of the housing and transportation sectors.

Zones 5, 6, and 7 had one sector with above-average and two with below-average potential. The number of counties in the three zones was relatively small, a total of 226, accounting for 12.7% of the 1771 counties. The proportions of Zones 5, 6, and 7 are 5.8%, 3.2%, and 3.7%, respectively. The overall performance of the counties was acceptable, with only one sector experiencing management problems. These counties should strive towards bottleneck breakthroughs in governance, actively improving low-carbon management in the weak sector and maintaining low-carbon management in other sectors to achieve sound management. Specifically, Zone 5 should improve the low-carbon management efficiency of the industrial sector, Zone 6 should strengthen the housing sector, and Zone 7 improve the low-carbon management level of the transportation sector.

There were only 39 counties in Zone 8, accounting for 2.2% of the counties, mainly distributed in the periphery of large cities and remote areas. All three sectors show below-average potential and sound low-carbon management. The counties in this zone should consolidate their existing low-carbon levels and management capabilities when forming policies. Likewise, they should help counties in other zones by sharing their experience.

### 4.4. Further Discussions and Suggestions

The results show that similar to the previous studies at the provincial and city level, low management efficiency and high potential are distributed in most counties in China and show significant spatial differences [21,23,55]. The spatial heterogeneity also differs in different management sectors. Moreover, from the analysis, it can be found that these heterogeneous patterns can all be explained, which demonstrates the rationality of the study results. In addition, our governance zoning found that the counties in urgent need of improving low-carbon management are mainly distributed in central China. It seems to be inconsistent with the efficiency and potential evaluation at both provincial and city levels, which indicates that Eastern China should focus more on management improvement compared with other regions [23,64]. However, it should be noted that these previous studies mainly compare the contribution of technology and management improvement to the emission reduction potential of the region, and the conclusions are based on the ratio of the management potential to the total potential. However, based on comprehensive analysis, due to the overall high efficiency and low emission reduction potential of Eastern China, its overall management improvement space is still less than that of the large area in central China, which is consistent with our research results.

Compared with the previous result from the perspective of driving impact and status evaluation, our results have certain differences. For instance, Zhang et al. (2021) found that the counties in Eastern China have the strongest impact from carbon emission drivers and can be the key area for carbon management [13]. However, this research, from the perspective of efficiency and potential, found that the wide range of counties in central China deserves more attention. It can be explained by the fact that the county economy and urbanization in Eastern China developed rapidly and drove more CO_2_ emissions. However, due to the higher development levels, the allocation efficiency of management factors and carbon emissions efficiency in Eastern China are relatively high, and the corresponding space for management improvement is not so large. On the contrary, the counties in central China blindly pursue development but do not pay attention to their own conditions and have no scientific management methods, resulting in low efficiency in low-carbon development, and exist the coexistence of management resource shortage and waste. In addition, unlike previous studies based on low-carbon development evaluation, at city-level believed that Western China should be the key management area [2]. Our research found that after considering the difference in resource endowment and development gap, there are still a large number of counties with small management improvement potential in Western China, and the room for improvement in Western China may not be so high.

Based on the analysis and discussion of research results, further suggestions can be explored.

The Chinese government should not ignore regional differences in counties and should adopt differentiated policy control levels and allocate low-carbon policy objectives. It is suggested that the decision should rely on the local conditions of counties and not unify within the whole country. In particular, attention should be paid to the counties with higher management improvement potential of all sectors (Zone1) and regard them as the key area of national governance. At the same time, we should carry forward the management experience of advanced counties and guide the learning and co-operation between counties. Counties with good low-carbon management of various sectors (Zone 8) can be selected as the national demonstration area and act as the overall goal of the low-carbon management improvement at the national level.
Local governments should formulate specific management improvement plans by focusing on the key low-carbon management sectors with higher improvement potential and refer to the counties with similar resource endowments to develop specific strategies. Based on the analysis of the efficiency and potential assessment results, the specific improvement suggestions are given as follows:
(1) For the counties where the industry is the key management improvement sector (Zone 1, 2, 3, 5), the management level of industrial structure should be strengthened, considering the industrial structure management level plays a decisive role in the spatial differentiation of the potential analysis among Chinese counties. Attention should be paid to confirming whether the industrial structure adjustment policy fit to the regional advantages. For example, as for counties with low development levels, the authorities should not blindly pursue secondary industrial development but refer to counties with relatively backward technical and development levels and encourage qualified areas to develop tourism, ecological agriculture etc.
(2) For the counties which take the housing sector as the key management improvement sector (Zone 1, 2, 4, 6) should reasonably control development intensity and strength the management level of municipal utility facilities. The reasonable control of residential density should refer to the counties with similar climate and topography conditions, considering the lighting and living quality. In addition, local governments should improve the allocation efficiency of municipal facilities. Moreover, the centralized infrastructure construction should give more residents access to clean energy and try to avoid energy loss. The specific configuration method can refer to the counties with similar climate and topography conditions.
(3) As for the counties with the transportation sector as the key improvement sector (Zone 1, 3, 4, 7), road traffic facilities should be rationally allocated, and functional space layout should be actively guided.Considering the high upgrading potential of provincial border areas, the accessibility and connectivity of trunk roads should be strengthened, especially in border areas. Road traffic planning should increase the coupling degree between urban population density, urban form and traffic organization. Moreover, public service facilities should ensure the residential demands, and the organization needs to improve equality and convenience to reduce long-distance travel efficiently. The specific plans can refer to counties with similar income levels and topography.

## 5. Conclusions

This paper aimed to help different regions formulate specific and realistic low-carbon management policies. It proposed an efficiency and potential evaluation method by incorporating specific low-carbon management sectors and indicators and considering various resource endowment conditions and barriers between regions. This paper took a large number of diverse Chinese counties as the research object, evaluated their low-carbon management efficiency and potential, and further determined their differentiated management zones and paths.

The evaluation results of the efficiency and potential of low-carbon management in the counties showed spatial heterogeneity. The main findings are as follows: (1) Regarding specific management efficiency, counties at the border of central and Western China and those in the southeast coastal areas showed higher efficiency in the industrial sector. Counties in Southern China showed higher efficiency in the housing sector, while counties in Northern China showed higher efficiency in the transportation sector; and (2) When it comes to the practical management potential, counties in remote areas need improvement in the industrial sector. Counties in central China showed high potential in the housing sector, while the ones bordering provinces need improvement in the transportation sector.

The results of efficiency and potential evaluation provide an important policy reference for low-carbon management. To further improve management, the areas were divided into eight governance zones where different policies were identified as conducive to national and local policy formulation and improvement. Among them, the counties most in need of management improvement are concentrated in central China. Management demonstration counties are mainly distributed around large cities and remote areas. The specific policy and operational significance can be summed up in the following way. Firstly, this paper is conducive to policy formulation at the national level. Based on the sectors in need of improvement, the paper established four policy control levels that can help the country allocate low-carbon policy objectives. Our research can also determine the key areas for national governance, i.e., the areas that need to be improved. Secondly, this paper identified the learning objectives at the national level. Next, our research can help local governments formulate specific low-carbon management policies according to local conditions. The local government can determine key low-carbon management sectors in the region according to the results of efficiency and potential. Furthermore, this paper gives local governments the most realistic reference for learning. Local governments can refer to the operation methods for the best efficiency of the sector in areas with similar resource endowments so as to improve the management efficiency of key sectors.

However, this study also has certain limitations which need to be improved in future research. This study used only cross-sectional data from 2015 rather than panel data from a longer period. Future research can incorporate a long-term or dynamic system analysis to investigate and monitor differential changes between regions.

## Figures and Tables

**Table 1:** The input and output elements of low-carbon management efficiency evaluation.

Administrative Sector	Input Elements		Output Elements
Industry	Industrial structure	Ratio of the added value of the primary industry to secondary industry	GDP output per unit of industrial carbon emission
		Proportion of the tertiary industry to GDP	
	Asset investment	Gross fixed capital formation	
Housing	Development intensity	Residential density	Residential population per unit of housing carbon emission
	Municipal utility facilities	Coverage rate of population with access to gas	
		Density of heating pipelines	
Transportation	Road traffic facilities	Density of road networks	Cover area per unit of carbon emission from transportation
		Sidewalk area per capita	
	Functional space layout	Matching ratio of educational facilities	
		Matching ratio of hospital accommodations	

**Table 2:** Grouping factors for low-carbon management potential evaluation.

Administrative Sector	Grouping Factors			
	Economic and Technological Development	Topography	Climate Conditions	Residents’ Income Levels
Industry	√			
Housing		√	√	√
Transportation		√		√

**Table 3:** Differentiated governance paths for each governance zone of Chinese counties.

Governance Zone	Governance Path			
	Key Management Sector			Policy Orientation
	Industry	Housing	Transportation	
Zone 1	√	√	√	Overall improvement
Zone 2	√	√		Coordinated improvement
Zone 3	√		√	
Zone 4		√	√	
Zone 5	√			Bottleneck breakthrough
Zone 6		√		
Zone 7			√	
Zone 8				Consolidate condition

## Data Availability

The data are taken from the High Spatial Resolution Greenhouse Gas Online Platform (https://wxccg.cityghg.com/geo; (accessed on 27 December 2022)), Chinese counties Construction Statistical Yearbook and the Chinese counties Statistical Yearbook. The data in this paper can be obtained from the authors.
